# Fe,Ni-Based Metal–Organic Frameworks Embedded in Nanoporous Nitrogen-Doped Graphene as a Highly Efficient Electrocatalyst for the Oxygen Evolution Reaction

**DOI:** 10.3390/nano14090751

**Published:** 2024-04-25

**Authors:** Panjuan Tang, Biagio Di Vizio, Jijin Yang, Bhushan Patil, Mattia Cattelan, Stefano Agnoli

**Affiliations:** 1 Department of Chemical Sciences, University of Padova, Via F. Marzolo 1, 35131 Padova, Italy; panjuan.tang@phd.unipd.it (P.T.);; 2 National Interuniversity Consortium of Materials Science and Technology (INSTM), 50121 Florence, Italy; 3 Consorzio Interuniversitario Reattività Chimica e Catalisi (CIRCC) Research Unit, University of Padova, 35131 Padova, Italy

**Keywords:** oxygen evolution reaction, electrocatalysts, metal–organic frameworks, N-doped graphene, hybrid materials

## Abstract

The quest for economically sustainable electrocatalysts to replace critical materials in anodes for the oxygen evolution reaction (OER) is a key goal in electrochemical conversion technologies, and, in this context, metal–organic frameworks (MOFs) offer great promise as alternative electroactive materials. In this study, a series of nanostructured electrocatalysts was successfully synthesized by growing tailored Ni-Fe-based MOFs on nitrogen-doped graphene, creating composite systems named MIL-NG-n. Their growth was tuned using a molecular modulator, revealing a non-trivial trend of the properties as a function of the modulator quantity. The most active material displayed an excellent OER performance characterized by a potential of 1.47 V (vs. RHE) to reach 10 mA cm^−2^, a low Tafel slope (42 mV dec^−1^), and a stability exceeding 18 h in 0.1 M KOH. This outstanding performance was attributed to the synergistic effect between the unique MOF architecture and N-doped graphene, enhancing the amount of active sites and the electron transfer. Compared to a simple mixture of MOFs and N-doped graphene or the deposition of Fe and Ni atoms on the N-doped graphene, these hybrid materials demonstrated a clearly superior OER performance.

## 1. Introduction

With the continuous growth of the world economy, fossil fuels such as oil, natural gas, and coal have been over-exploited as primary energy sources, causing serious environmental problems such as pollution and systematic negative impacts on the climate [1,2]. To overcome this, the development of a cleaner and sustainable energy infrastructure based on renewable sources and hydrogen as the primary energy vector is a promising route [3,4,5]. Thence, the demand for hydrogen and its exploitation in different sectors are increasing, and we need efficient methods for its large-scale production [6]. In this context, electrocatalytic water splitting is recognized as the most promising technology for producing sustainable hydrogen [7,8]. The development of catalysts with high activity [9,10] at low costs [11] for the oxygen evolution reaction (OER) is one of the most critical bottlenecks that still limits an important part of the electrolyser market [12]. Precious and critical metal oxides (such as RuO_2_ and IrO_2_) [13,14] have traditionally been used as electrocatalysts for the OER, however, their prohibitive cost prevents their large-scale production and commercial application. Transition-metal-based catalysts, such as oxides/hydroxides nanoparticles [15], perovskite [16,17], and layered double hydroxides (LDHs) [18,19,20], are extremely promising given their good activity, large abundancy, and good stability under alkaline conditions. Although transition-metal-based electrocatalysts display interesting performances, they still cannot compare with traditional noble-metal-based materials. Thence, doping and alloying have emerged as efficient methods for increasing the chemical activity, leading to the identification of mixed metal oxides/hydroxides materials (e.g., NiFeO_x_ [21,22] and Ni/Co/Fe layered double hydroxides [23,24]) as the most viable electrocatalysts for the OER.

On the other hand, metal–organic frameworks (MOFs), i.e., microporous crystalline materials made of metal nodes connected by organic building blocks, have been recently proposed as catalytic materials for energy conversion, given their large surface area, good thermal stability, high porosity, tunable functionalities, and large availability of open metal sites [25,26,27,28,29]. Materials Institute Lavoisier (MIL) compounds are a class of crystalline MOFs composed of different trivalent metal cations and carboxylic acid ligands [30,31,32]. Fe-, Ni-, and Co-based MOFs of the MIL family have been investigated as catalysts for the OER and extremely good results were obtained by the action of the redox chemistry of the metal nodes and tailoring the pore environment [33,34,35]. However, single-phase MOF materials suffer a very fundamental shortcoming for electrocatalysis, which is a poor electronic conductivity. Several literature works have reported solutions to this problem by supporting catalytically active MOFs on highly conductive and mechanically stable materials such as graphene oxide (GO) and nitrogen-doped graphene (NG) [36,37,38]. 

In recent years, GO [39] and NG [40] materials have captured a great deal of attention as applied materials owing to their affordable large-scale production and interesting properties, including large surface area, good electrical conductivity, and strong mechanical strength. The highly adaptable 2D structure of graphene and its easy chemical functionalization [41,42] provide a variety of active sites for its combination with other materials [43]. Therefore, the combination of graphene with MOFs represents an effective strategy for building high-performing nanocomposites that synergistically combine the best properties of the two moieties, as demonstrated by some works that have clearly proven the benefit in the incorporation of graphene to improve the electrocatalytic and photocatalytic properties of semiconducting MOFs [44,45,46,47]. 

Having in mind the OER in alkaline conditions as the target reaction, it is natural to study Fe-Ni-based compounds. Indeed, Ni-Fe-based oxides/oxyhydroxides have emerged as highly promising non-noble-metal-based electrocatalysts for the OER, with numerous studies showcasing the ability to modify their physicochemical properties by adjusting the stoichiometry of the Ni and Fe ions [48,49]. In particular, the effect of the incorporation of Fe ions into Ni catalysts can improve electron conductivity and reaction kinetics due to their optimal bond energetics for the adsorption of OER intermediates [50]

In this paper, hybrid nanoarchitectures with a tailored chemical composition and morphology, consisting of mixed metal MIL-126 supported on NG, were prepared through a solvothermal route by controlling the coordination reaction between Fe/Ni ions, biphenyl-4,4′-dicarboxylic acid (H_2_bpdc), and NG. Particularly relevant is the “modulator approach” widely adopted in MOF synthesis, where a monocarboxylic acid is used to regulate the kinetics and morphology of the MOF growth [51,52]. In general, modulators facilitate the formation of metal clusters and slow down the crystal growth rate, thus avoiding the fast precipitation of amorphous products. Here, we used acetic acid (HOAc) as the modulator to control the nucleation density and growth rate of the MOF nanoparticles on the surface of NG to optimize the electrocatalytic activity of the resulting nanocomposite, noted as MIL-NG-n. The fabricated materials with different HOAc contents were extensively characterized, focusing on their structural and electrochemical behaviors. The evolution of the morphology, structure, and chemical composition of the MIL-NG-n along with variations in the acetic acid were investigated by powder X-ray diffraction (PXRD), scanning electron microscopy (SEM), energy-dispersive X-ray spectroscopy (EDX), transmission electron microscopy (TEM), X-ray photoemission spectroscopy (XPS), and the Brunauer−Emmett−Teller (BET) method. The electrocatalytic behavior of the samples was tested in a three-electrode electrochemical cell using cyclic voltammetry (CV) and linear sweep voltammetry (LSV). 

Importantly, the results indicated that the NG-MOF hybrids could promote the OER and induce higher electrocatalytic activity than the parent MOFs. When employed as an OER catalyst, the MIL-NG-3 had a low overpotential of 240 mV (at a current density of 10 mA cm^−2^) and low Tafel slope in alkaline solution, which outperforms most traditional crystalline inorganic materials.

## 2. Materials and Methods

### 2.1. Chemicals

All chemicals employed in this study were of analytical grade and were used without further purification. Biphenyl-4,4′-dicarboxylic acid (H_2_bpdc), nickel(II) nitrate hexahydrate (Ni(NO_3_)_2_·6H_2_O), iron(III) chloride hexahydrate (FeCl_3_·6H_2_O), potassium hydroxide (KOH), Nafion^®^ 117 ∼5% in ethanol and water, ethanol (CH_3_CH_3_OH, 99.7%), dimethylformamide (DMF), N-methyl-2-pyrrolidone (NMP), acetic acid (HOAc), fluorinated graphite, and ammonium hydroxide solution (28–30%) were provided by Merck & Co Inc., (Rahway, NJ, USA). Deionized water was supplied by a Millipore System.

### 2.2. Physical Chemical Characterizations

Powder X-ray diffraction (PXRD) patterns were recorded on a PANalytical PW3040/60 X’Pert PRO MPD (Malvern Panalytical B.V, Almelo, The Netherlands) X-ray diffractometer with Cu-Ka radiation (1.5418 Å). SEM images were obtained by a Zeiss Supra VP35 (Carl Zeiss SMT, Wetzlar, Germany) scanning electron microscope and an FEG-FIB Tescan Solaris with an EDX detector Ultim Max 65 (Oxford Instrument, Abingdon-on-Thames, UK). TEM images were obtained using an FEI Tecnai 12 operating under an accelerating voltage of 200 kV. The surface area and pore size characteristics of the synthesized samples were analyzed by N_2_ adsorption and desorption measurements using an ASAP 2020 Micromeritics surface area and porosity analyzer (Norcross, GA, USA). Before the nitrogen adsorption/desorption measurements were performed, all samples were dried at 120 °C under vacuum for 12 h. Raman measurements were performed with a ThermoFisher DXR Raman microscope (Waltham, MA, USA) using a laser with an excitation wavelength of 780 nm (1 mW) and a 50× objective (Olympus, Tokyo, Japan). XPS spectra were acquired using the Mg Kα line (hν = 1253.6 eV) and a pass energy of 20 eV.

### 2.3. Electrochemical Measurements

OER tests were performed in a three-electrode cell with an Autolab potentiostat (Metrohm Group, Utrecht, The Netherlands), using a graphite counter electrode and an Ag/AgCl/saturated KCl reference electrode. The catalyst ink for the electrochemical analysis was prepared as follows: 4 mg of active powder was dispersed in H_2_O (490 μL), DMF (490 μL), and a 5 wt % Nafion dispersion solution (20 μL), then sonicated for 1 h in an ultrasonic bath. After sonication, 8.5 μL of the mixture was drop cast on the glassy carbon electrode, which was previously polished with Al_2_O_3_ paste and rinsed with deionized water/ethanol. At the end of the procedure, a thin and mechanical robust catalyst layer with a loading of 0.2 mg cm^−2^ and a fixed area of 0.159 cm^2^ was obtained. Before the OER performance was tested, the catalysts were activated via CV scans at 100 mV s^−1^ until they were stable. Linear sweep voltammetry was acquired by sweeping potentials from 0.93 to 1.9 V (vs. the Reversible Hydrogen Electrode (RHE)) with a forward scan rate of 5 mV s^−1^ in 0.1 M KOH (pH 12.7). The current density was normalized to the geometrical surface area of the working electrode. All the polarization curves presented were corrected for the cell resistance. All the potentials reported in this study were referred against the RHE using Equation (1).

E (RHE) = E(Ag/AgCl) + (0.059 × pH) + 0.183(1)

### 2.4. Synthesis of NG

A volume of 13 mL of degassed NMP was added to 120 mg of fluorinated graphite (FG) under a N_2_ atmosphere, and the mixture was sonicated for 4 h. Then, NH_3_, produced by heating 200 mL of ammonium hydroxide solution at 60 °C and dehydrating the resulting vapors with a KOH pad, was bubbled in the suspension kept at 0 °C with an ice bath for 2 h. Finally, the mixture was transferred to a cold Teflon-lined autoclave and heated from room temperature to 140 °C with a heating rate of 1 °C/min, where it was kept at the final temperature for 60 h. The Teflon-lined autoclave was then allowed to reach room temperature. After cooling down, the mixture was centrifuged and washed with DMF (50 mL × 2), a mixture of DMF and water (50 mL × 2), a mixture of MeOH and water (50 mL × 2), MeOH (50 mL × 2), and acetone (50 mL × 2).

### 2.5. Synthesis of Bimetallic (Fe,Ni)-MIL-126

The (Fe,Ni)-MIL-126 was synthesized via a solvothermal route. H_2_bpdc (22.4 mg) was dissolved in DMF (3 mL) under ultrasonication, while FeCl_3_·6H_2_O (27 mg) and Ni(NO_3_)_2_·6H_2_O (46.6 mg) were dissolved in 6 mL of DMF and 300 µL of CH_3_COOH (5 mmol). The solution was poured into a Teflon-lined autoclave and heated to 150 °C with a heating rate of 5 °C per min, where it was maintained at this temperature for 15 h. After cooling down to room temperature, the mixture was centrifuged and washed with DMF and MeOH multiple times.

### 2.6. Synthesis of MIL-NG-n Hybrid 

**Figure 1:**
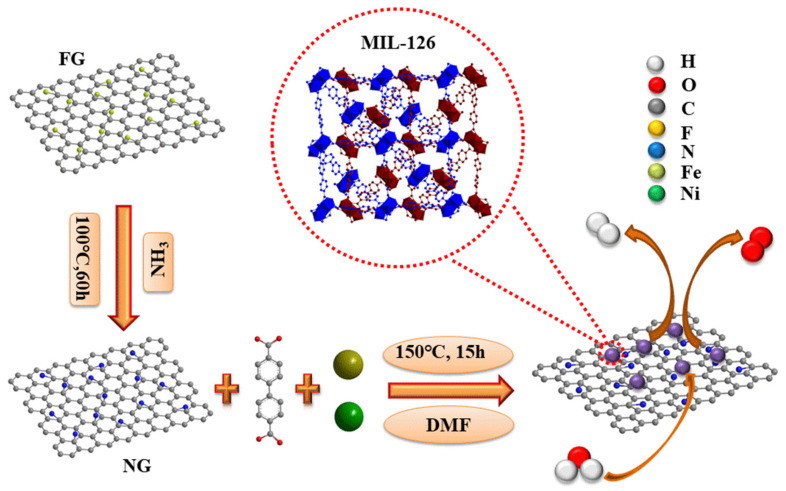
Schematic illustration of the synthesis of MIL-NG-n hybrids.

The schematics of the synthesis of the MIL-NG-n hybrids are reported in Figure 1. To prepare the MIL-NG-n hybrids, an amount of 5 mg of NG was added to 9 mL of DMF and then kept at room temperature for 1 h until a homogeneous suspension was obtained. Then, FeCl_3_·6H_2_O (27 mg) and Ni(NO_3_)_2_·6H_2_O (46.6 mg) were added, and the suspension was further sonicated for 1 h and then stirred for 12 h. Afterward, different quantities of CH_3_COOH (HOAc) (see Table 1) and H_2_bpdc (22.4 mg) were added to the mixture. After sonicating the solution for 1 h, the reaction vessel was sealed and heated up to 150 °C with a heating rate of 5 °C/min, where it was kept at the final temperature for 15 h. After cooling down, the mixture was centrifuged and washed with DMF (50 mL × 3) and MeOH (50 mL × 3).

### 2.7. Synthesis of Bimetallic (Fe,Ni)-NG

To synthesize the (Fe,Ni)-NG hybrid, an amount of 5 mg of NG was added to 9 mL of DMF. Then, the mixture was sonicated at room temperature for 1 h until a homogeneous suspension was obtained. Then, FeCl_3_·6H_2_O (27 mg) and Ni(NO_3_)_2_·6H_2_O (46.6 mg) were added to the mixture, sonicated for 1 h, and stirred for 12 h. Then, the reaction vessel was sealed and heated up to 150 °C with a heating rate of 5 °C/min, where it was kept at the final temperature for 15 h. After cooling down, the mixture was centrifuged and washed with DMF and MeOH multiple times.

### 2.8. Synthesis of (Fe,Ni)-MIL-126-NG-mix

To prepare the (Fe,Ni)-MIL-126-NG-mix, i.e., the physical mixture of the NG and the MOF, 2 mg of NG and 2 mg of (Fe,Ni)-MIL-126 were mixed by extensive grinding in an agate mortar.

## 3. Results and Discussion

The formation process of the MIL-NG-n hybrids is illustrated in Figure 1, while Table 1 reports the different amounts of modulator (HOAc) used in the synthesis, which was tuned from 0 to 3 mol. The in situ growth of MOFs on functionalized graphene is one of the most extensively used strategies for MOF/graphene hybrids [31,45]. This method ensures the uniform growth of MOFs on graphene, fostering a strong interaction between the two materials. This interaction arises from the abundant functional group of NG, and N is about 20% of the C, which can coordinate metal ions during the synthesis and facilitate the in situ nucleation and growth of MOF nanocrystals. In this paper, we specifically used NG deriving form fluorinated graphite, because this special synthesis route allows for achieving a very high level of nitrogen doping, with pyridinic and pyrrolic groups in an approximated ratio of 2:1 (see Supporting Information, Figures S1 and S2).

**Figure 2:**
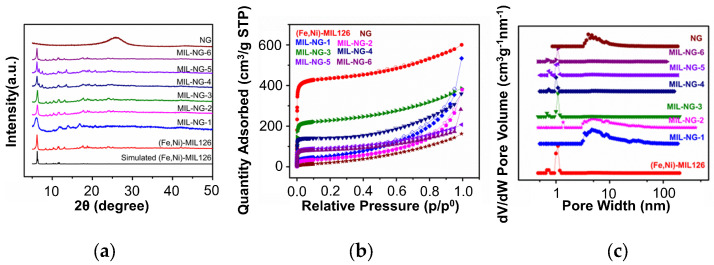
(**a**) XRD, (**b**) BET, and (**c**) pore size distribution patterns of as-prepared NG-MIL-n, (Fe,Ni)-MIL-126 and NG samples.

The crystallographic structures of the as-prepared (Fe,Ni)-MIL-126 and the MIL-NG-n nanocomposites were examined by PXRD (Figure 2a). The (Fe,Ni)-MIL-126 nanocomposite showed a diffraction pattern compatible with the two-fold interpenetrated structure based on Fe^3+^ ions and bpdc linkers reported by Serre et al. in 2012 (MIL-126(Fe), CCDC code MIBMER) [30]. The well-defined diffraction peaks confirm the high crystallinity of (Fe,Ni)-MIL-126 [31]. The MIL-NG-3, MIL-NG-4, MIL-NG-5, and MIL-NG-6 nanocomposites showed similar diffraction peaks as (Fe,Ni)-MIL-126, suggesting that the crystallinity of (Fe,Ni)-MIL-126 was not disrupted by the interaction with NG, i.e., the links between the organic bridges and metal ions were not interrupted.

However, comparing the XRD pattern of (Fe,Ni)-MIL-126 with those of MIL-NG-1 and MIL-NG-2, the characteristic diffraction peaks are much broader, indicating a lower crystallinity of the materials. Probably, in this case, the low concentration of modulator produces a non-uniform crystal growth allowing for late nucleation events [53]. Moreover, it has been reported that, in the absence of a modulator, a poorly crystalline MIL-88D phase can be obtained as well [54]. On the other hand, with the increase in HOAc from 1 mmol to 3 mmol, the characteristic peaks of the target MOF became well-defined, indicating that a minimum quantity of modulator is necessary to promote crystallization. Nonetheless, there was a marked decrease in the MOF yield indirectly visible by a reduction in the PXRD intensity. These findings are consistent with results reported by Forgan et al., who demonstrated that coordination modulation typically slows down the kinetics, allowing the formation of the most stable thermodynamic compounds to be reached (i.e., two-fold interpenetrated MIL-126) [54].

The porous features and BET specific surface areas of (Fe,Ni)-MIL-126, NG, and all the MIL-NG-n hybrids were investigated by nitrogen isothermal adsorption/desorption measurements at 77 K. The BET analysis results are reported in Table 2. Figure 2b shows that the (Fe,Ni)-MIL-126, MIL-NG-3, MIL-NG-4, MIL-NG-5, and MIL-NG-6 hybrid samples exhibited a typical type I isotherm, confirming that all the composites were predominantly microporous materials, whereas the NG, MIL-NG-1, and MIL-NG-2 hybrids presented a typical type IV adsorption isotherm shape with a hysteresis loop, demonstrating the existence of mesoporous characteristics. Compared with the MIL-NG-n hybrid, the pure (Fe,Ni)-MIL-126 had much better N_2_ adsorption capacity, particularly below a pressure of P/P_0_ = 0.1. The increase in the quantity of HOAc induced a non-monotonic trend of the surface area: for low or no quantity, i.e., from MIL-NG-1 to MIL-NG-3, there was a clear increase in porosity, whereas a significant decrease was observed at higher concentration. A similar trend is discussed above for the XRD data, with an increase in crystallinity from MIL-NG-1 to MIL-NG-3 followed by a decrease in the MOF yield from MIL-NG-3 to MIL-NG-6. The pore volumes and pore size distributions from the N_2_ isotherms were obtained by using a nonlinear density functional theory model. The pores size in (Fe,Ni)-MIL-126, MIL-NG-3, MIL-NG-4, MIL-NG-5, and MIL-NG-6 were mainly centered at 1 nm, indicating the presence of micropores, consistent with the N_2_ adsorption isotherm in Figure 2c. The MIL-NG-1, MIL-NG-2, and NG also showed some mesoporosity, with most of the pore sizes in the 5 nm range. 

Among all the MIL-NG-n hybrids, MIL-NG-3 exhibited a much higher surface area, which is a favorable aspect for electrocatalysis, given the larger exposure of catalytic active sites and improved resulting mass transport.

**Figure 3:**
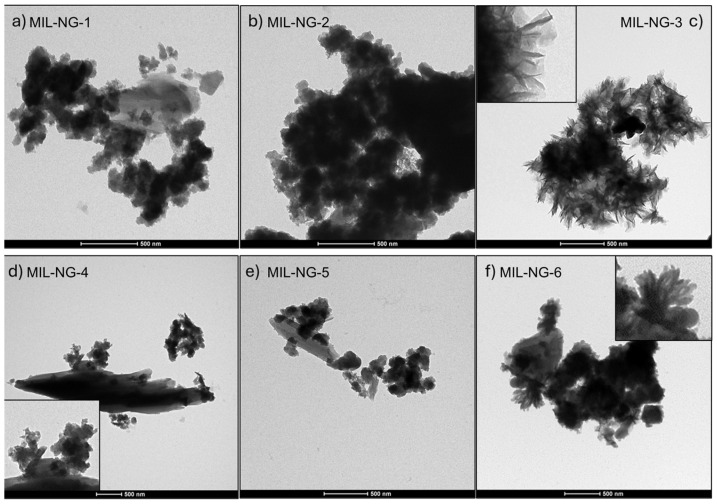
TEM images of as-prepared (**a**) MIL-NG-1, (**b**) MIL-NG-2, (**c**) MIL-NG-3, (**d**) MIL-NG-4, (**e**) MIL-NG-5, and (**f**) MIL-NG-6, scale bars 500 nm.

To visualize how the (Fe,Ni)-MIL-126 and NG components were assembled into the hybrid materials, we performed SEM and TEM measurements. The SEM images of the NG-MIL-n samples are reported in Figures S3 and S4 along with EDX mapping and spectra. The EDX analysis proves the co-presence NG and MOF in the same position detectable from N and C signals, mainly related to NG, and Fe and Ni absorption peaks, associated with the MOF [52]. The structures of the MIL-NG-n samples prepared with different amounts of modulator are shown in Figure 3a–f, where it can be clearly seen that (Fe,Ni)-MIL-126 nanoparticles, in a range from 50 to 200 nm, are dispersed quite homogenously on NG flakes. However, as the content of HOAc changes, the morphology of the resulting MOF shows no clear changes at these magnifications. In Figure S5. the TEM image of the bare NG supports is reported for comparison.

The materials’ characterization demonstrates that the obtained MIL-NG-n hybrids possessed the necessary characteristics for a highly efficient OER electrocatalyst with a highly dispersed ensemble of exposed metal centers, i.e., (Fe,Ni)-MIL-126, in close contact with a highly conductive material, i.e., NG, for fast electron transfer.

**Figure 4:**
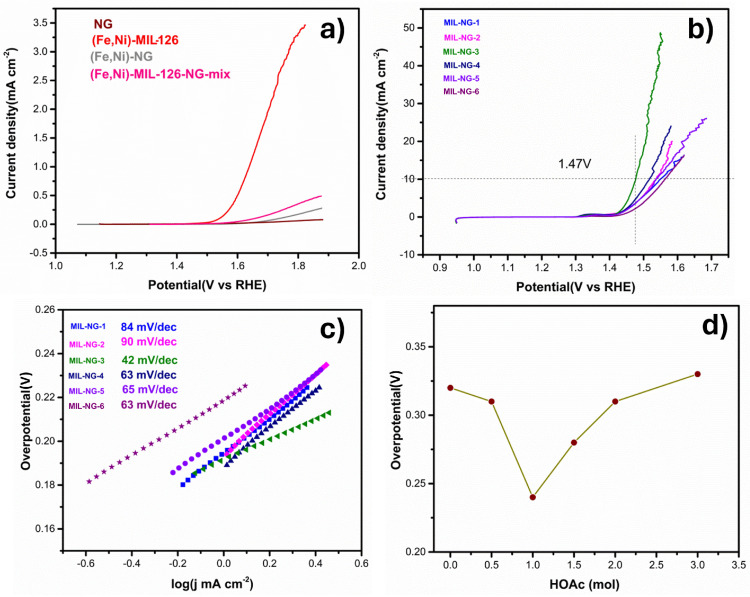
OER current densities of glassy carbon (GC)-supported in 0.1 M KOH at 5 mV s^−1^: (**a**) LSV curves of NG, (Fe,Ni)-MIL-126, bimetallic (Fe,Ni)-NG, and (Fe,Ni)-MIL-126-NG-mix, (**b**) LSV curves of MIL-NG-n, (**c**) Tafel plots of MIL-NG-n, and (**d**) the overpotential as a function of the molar of HOAc.

To prove that the hybrid materials had superior electrocatalytic activity, firstly, we compared the bare carbon support, the pure (Fe,Ni)-MIL-126, the physical mixture of NG and (Fe,Ni)-MIL-126 ((Fe,Ni)-MIL-126-NG-mix), and a composite produced by the direct metalation of the nitrogen centers of NG with Ni and Fe metal cations ((Fe,Ni)-NG). As reported in Figure 4a the NG, bimetallic (Fe,Ni)-NG, and (Fe,Ni)-MIL-126-NG-mix showed low OER activity, whereas for (Fe,Ni)-MIL-126, the electroactivity started to become significant. This indicates that the metal centers are obviously essential for catalysis, however, to achieve a good performance, it is also necessary to guarantee that they are efficiently utilized, i.e., directly exposed to the electrolyte. The other important point for high electrochemical activity is also the ability to transfer electrons, which is possible only if the MOF, which is rather electrically resistive and is efficiently connected to a highly conductive support. Indeed, any MIL-NG-n hybrid electrocatalyst (Figure 4b) showed much higher activity than all the samples reported in Figure 4a, demonstrating the synergistic effects of NG and (Fe,Ni)-MIL-126. 

In Figure 4a, when comparing the performances of pure (Fe,Ni)-MIL-126 and the physically mixed composite 50% in weight with NG ((Fe,Ni)-MIL-126-NG-mix), it seems that the OER performance was hindered by the NG when the two components were simply mixed. Specifically, the current density at 1.9 V vs. RHE of the (Fe,Ni)-MIL-126-NG-mix was about seven times lower than that of the pure MOF. This suggests that NG may have covered some metallic centers of the MOF, or in any case, it did not significantly help the activity of the MOF, as it was not electrically connected to it. These observations underscore the critical importance of the synthesis approach proposed in our work [45,55], which facilitates an intimate contact between MOF and graphenic materials. Hybrid materials are not achievable through simple mixing or metal doping, and only when effectively formed do they have enhanced conductivity and a significantly increased active surface area.

To understand the modulator effect, the overpotential necessary to reach 10 mA cm^−2^ for different hybrids was plotted in Figure 4d. MIL-NG-3 was the best-performing electrode with an overpotential of 240 mV, which was significantly lower compared to the other non-hybrid electrodes in Figure 4a, which did not even reach 10 mA cm^−2^ at an overpotential of 670 mV. Moreover, at an overpotential of 310 mV, the current densities of MIL-NG-3 could reach 40 mA cm^−2^, which was 2.6 and 4 times more than MIL-NG-4 and MIL-NG-5, respectively.

Furthermore, different concentrations of HOAc exhibited a discernible pattern in terms of catalytic activity, reaching a peak value at 1 mol of HOAc (MIL-NG-3). Similar trends were observed in PXRD, with well-defined MOF peaks at the critical minimum modulator quantity for MIL-NG-3 (refer to Table 1), and in BET, showing the larger surface area of the hybrids for MIL-NG-3 (refer to Table 2). The above results indicate that HOAc can favor high OER activity for an increase in MOF crystallinity, and a critical quantity of modulator is necessary to have a diffuse nucleation and controlled crystal growth.

The OER kinetics were analyzed by Tafel plots, as shown in Figure 4c. A low value of the Tafel slope is highly advantageous, as it allows for the achievement of a high catalytic current density at low applied potentials. The obtained Tafel slopes are summarized in Table 3. The results demonstrate that MIL-NG-3 exhibited rapid kinetics for OER compared to other MIL-NG-n hybrids, further explaining the enhancement of OER properties. Our best catalyst resulted in being one of the best-performing MOF–graphene hybrids in the literature, see Table 4.

**Figure 5:**
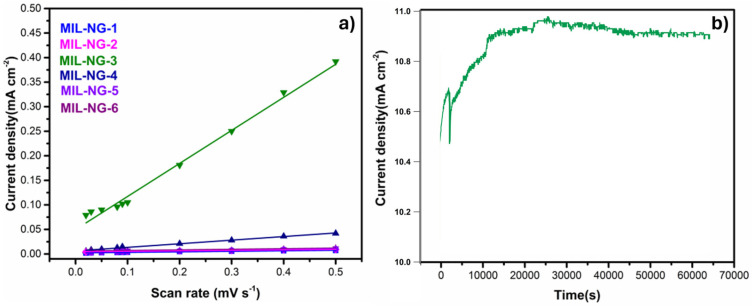
(**a**) Plot of current density vs. scan rate. (**b**) Chronoamperometric responses of NG-MIL-3 catalysts obtained at 1.47 V (vs. RHE) in 0.1 M KOH solution.

To better investigate the enhancement of OER activity for NG-MIL-3, electrochemically active areas (ECSA) can be evaluated from the electrochemical double-layer capacitance (Cdl) by conducting variable scan rate CV in the non-Faradaic region, see Figure 5a and Table 3. MIL-NG-3 had the highest Cdl value and the best OER performance, suggesting that its higher activity was linked to the more electrochemical available active sites at the solid–liquid interface, which was also linked to the higher surface area detectable by BET, see Table 2. The principal electrochemical key parameters of MIL-NG-n are summarized in Table 3.

Stability is also an important parameter for evaluating the properties of an electrocatalyst. In Figure 5b, the long-term durability of MIL-NG-3 is reported. A test was performed using current-time chronoamperometry at a constant voltage of 1.47 V (vs. RHE) in 0.1 M KOH. The obtained current density increased slightly in the first 4 h, rising from 10.4 to about 10.9 mA cm^−2^, and then remained quite constant for the following 13.5 h, indicating a great overall stability. 

**Figure 6:**
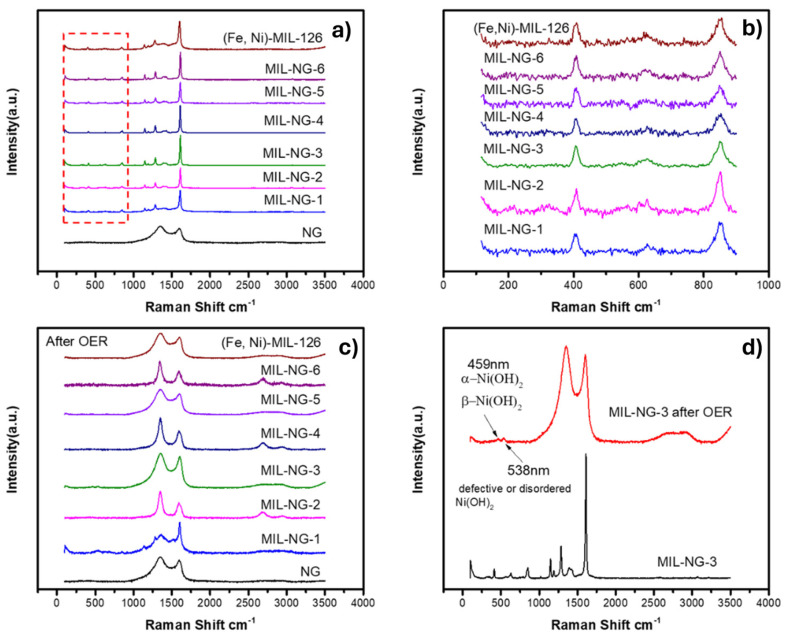
Raman spectra of the prepared (**a**) MIL-NG-n, (Fe,Ni)-MIL-126, and NG fresh samples, (**b**) detailed spectra from 100 to 900 cm^−1^ in (**a**), (**c**) MIL-NG-n, (Fe,Ni)-MIL-126, and NG after OER samples, and (**d**) comparison of MIL-NG-3 before and chronoamperometry in OER.

The PXRD investigation of the materials after the electrochemical was inconclusive, showing a substantial amorphization but without the identification of new ordered phase, so Raman spectroscopy was employed to understand the effect of the OER on the catalyst. The Raman signatures of NG, (Fe,Ni)-MIL126, and MIL-NG-n are shown in Figure 6. For NG, the two peaks are ascribed to the characteristic D-band and G-band of carbonaceous materials [56]. The Raman spectra of the MIL-NG-n hybrid materials show no significant differences compared with (Fe,Ni)-MIL-126. The MIL-NG-3 Raman spectra after 18 h chronoamperometry in the OER show peaks indicative of Ni(OH)_2_ phases [57]. This suggests a potential transformation of the materials, likely occurring within the first 4 h, coinciding with the visible increase in OER performance, see Figure 4b. An XPS analysis post-chronoamperometry in Figure S6 confirms the presence of Ni(OH)_2_/NiOOH and FeOOH. The notable activity and stability in the OER can, therefore, be ascribed to the existence of these materials; indeed, it is well-established that they are among the most used and stable materials for the OER in an alkaline medium [24,58]. 

Indeed, the limited stability of MOFs in strongly alkaline solutions and their restructuring in metal oxo-hydroxide have eventually emerged in the literature [59], but this apparent problem can also offer new synthetic strategies, as demonstrated in a recent seminal paper [60], where extremely active OER catalysts were obtained by sandwiching metal hydroxide nanosheets in between organic layers of pi-staked organic linkers, forming a so called metal hydroxide–organic framework. We can hypothesize that this is what happened in our materials, although in a less ordered and controlled way: the metal nodes rearranged into mixed metal oxo-hydroxide that remained confined within an organic shell made by the organic linker and the NG. 

Finally, it should be highlighted that our synthesis protocol is simple, safe, cost-effective, and leads to catalysts with excellent activity. Therefore, with further durability studies, they can be considered for application in the development of real electrolyzers.

**Table 3:** Summary of electrochemical OER performance data of MIL-NG-n.

Sample	Tafel Slope/mV dec^−1^	Overpotential at 10 mA cm^−2^/mV	Cdl/mF cm^−2^
MIL-NG-1	84	320	0.01
MIL-NG-2	90	310	0.009
MIL-NG-3	42	240	0.67
MIL-NG-4	63	280	0.07
MIL-NG-5	65	310	0.012
MIL-NG-6	63	330	0.015

**Table 4:** Comparison of OER performances of Ni-Fe based materials and MOF-graphene composites.

Components	E_OER_ (V) at 10 mA cm^−2^	Tafel mV·dec^−1^	Refs.
MIL-NG-3	1.47	42	/
MIL-126(FeNi)-700	2.0		[61]
Fe_1_Ni_2_-BDC	1.49	35	[26]
Fe/Ni/Co(Mn)-MIL-53/NF	1.45	53.5	[35]
Fe^2+^-NiFe LDH	1.479	40.4	[62]
NiFeV LDHs	1.422	42	[63]
NiFe LDHs Nanosheets	1.459	62.9	[64]
flame-engraved NiFe-LDH	1.48	69	[65]
Fe−Ni−P/rGO-400	1.47	63	[66]
Ni_2_P@C/GO (NiBTC)	1.515	44	[67]
NGO/Ni7S6 (Ni-MOF-74)	1.61	45	[68]
Ni-NiO/N-rGO	1.47	43	[28]
Ni-MOF-600	1.6	66	[69]
CoP/rGO-400 (ZIF 67)	1.57	66	[70]

## 4. Conclusions

We synthesized a series of highly active Ni,Fe-based MOFs within NG using meticulous NG-templated MOF growth with a ‘modulator’ approach. The resulting hybrids, called MIL-NG-n, exhibited an exceptional OER performance, with their physical–chemical properties showing a complex trend with respect to the modulator quantify. The outstanding OER performance was attributed to synergistic effects of the MOF’s large surface area and NG conductivity, enhancing the electrocatalytic activity, electrical conductivity, mass transfer, and overall activity. Among the synthesized catalysts, MIL-NG-3 was the most efficient, with an exceptional 240 mV overpotential to reach 10 mAcm^−2^ and a low Tafel slope of 42 mV dec^−1^. This work shows a significant advancement in the field, serving as a valuable reference for preparing electrocatalysts for OER applications without the use of critical raw materials. This approach opens up new avenues for preparing sustainable and high-performance electrocatalysts, contributing to green energy technologies. 

## Figures and Tables

**Table 1:** HOAc quantity used in the synthesis of the different nanocomposites. Other conditions are: 5 mg NG; 0.10 mmol FeCl_3_·6H_2_O; 0.16 mmol NiCl_2_·6H_2_O; 0.10 mmol H_2_bpdc; 9 mL of DMF.

	MIL-NG-1	MIL-NG-2	MIL-NG-3	MIL-NG-4	MIL-NG-5	MIL-NG-6
HOAc (mmol)	/	0.5	1	1.5	2	3

**Table 2:** BET method data.

Sample	Type of Porosity	Specific Surface Area (m^2^/g)	Pore Size (nm)
NG	IV type and hysteresis loop, mesoporous	92	5
(Fe,Ni)-MIL-126	I type, microporous	1720	1
MIL-NG-1	IV type and hysteresis loop, mesoporous	148	5
MIL-NG-2	IV type and hysteresis loop, mesoporous	196	5
MIL-NG-3	I type, microporous	884	1
MIL-NG-4	I type, microporous	595	1
MIL-NG-5	I type, microporous	352	1
MIL-NG-6	I type, microporous	285	1

## Data Availability

Data are contained within the article.

## Supplementary Materials

The following supporting information can be downloaded at: https://www.mdpi.com/article/10.3390/nano14090751/s1, Figure S1: NG Fourier Transformed Infrared (FT-IR), prepared on KBr disk. Spectrum recorded with a Nicolet Nexus FT-IR spectrometer. The characteristic NG bands of C-N (~1160 cm^−1^) and C=C (~1550 cm^−1^) vibrations are visible.; Figure S2: NG not-monochromatic X-ray photoemission spectroscopy (XPS) lines of C1s and N1s, deconvoluted into single chemically shifted components. Figure S3: SEM images of as-prepared (a) MIL-NG-1, (b) MIL-NG-2, (c) MIL-NG-3, (d) MIL-NG-4, (e) MIL-NG-5, (f) MIL-NG-6. Figure S4: SEM EDX mapping of MIL-NG-3 and EDX spectrum. Figure S5: TEM image of NG, scale bar 100 nm. Figure S6: Ni 2p_3/2_ and Fe 2p XPS lines of MIL-NG-3 after chronoamperometry in OER indicating the presence of Ni(OH)_2_/NiOOH and FeOOH.
