# Supplementary material for: Fe,Ni-Based Metal–Organic Frameworks Embedded in Nanoporous Nitrogen-Doped Graphene as a Highly Efficient Electrocatalyst for the Oxygen Evolution Reaction
Source: Nanomaterials (Basel). 2024 Apr 25;14(9):751. doi: 10.3390/nano14090751 (PMC11085937; doi:10.3390/nano14090751)
Supplement: Supplementary file 1 [file nanomaterials-14-00751-s001.zip › nanomaterials-2977490-supplementary.pdf]

Supplementary Information

Fe,Ni-Based Metal–Organic Frameworks Embedded in Nanoporous Nitrogen-Doped Graphene as a Highly Efficient Electrocatalyst for the Oxygen Evolution Reaction

Panjuan Tang [1,†], Biagio Di Vizio [1,†], Jijin Yang [1], Bhushan Patil [1], Mattia Cattelan [1,2,3,*] and Stefano Agnoli [1,2,3,*]

[1] Department of Chemical Sciences, University of Padova, Via F. Marzolo 1, 35131 Padova, Italy; panjuan.tang@phd.unipd.it (P.T.)

[2] National Interuniversity Consortium of Materials Science and Technology (INSTM), 50121 Florence, Italy

[3] Consorzio Interuniversitario Reattività Chimica e Catalisi (CIRCC) Research Unit, University of Padova, 35131 Padova, Italy

* Correspondence: mattia.cattelan@unipd.it (M.C.); stefano.agnoli@unipd.it (S.A.); Tel.: +39-0498275845 (M.C.); +39-0498275167 (S.A.)

† These authors have equally contributed to this work.

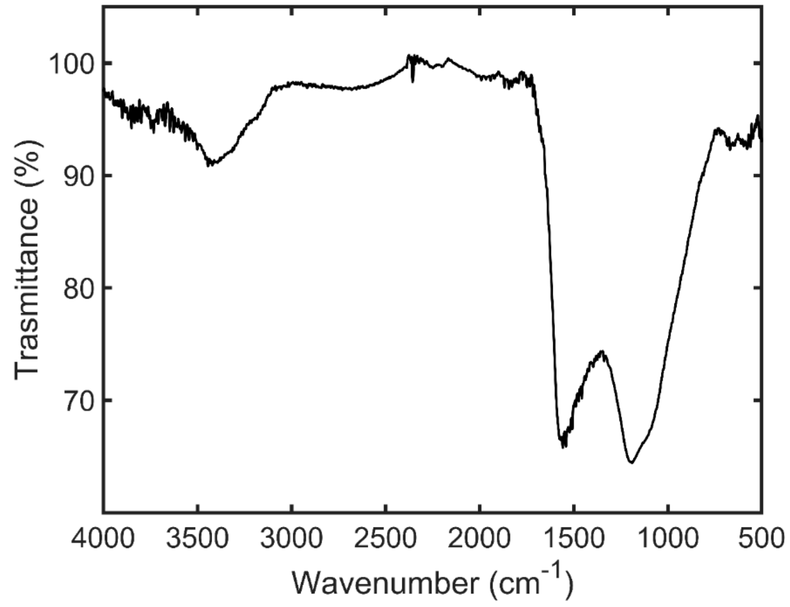

Figure S1: NG Fourier Transformed Infrared (FT-IR), prepared on KBr disk. Spectrum recorded with a Nicolet Nexus FT-IR spectrometer. The characteristic NG bands of C-N (~1160 cm^{-1}) and C=C (~1550 cm^{-1}) vibrations are visible.

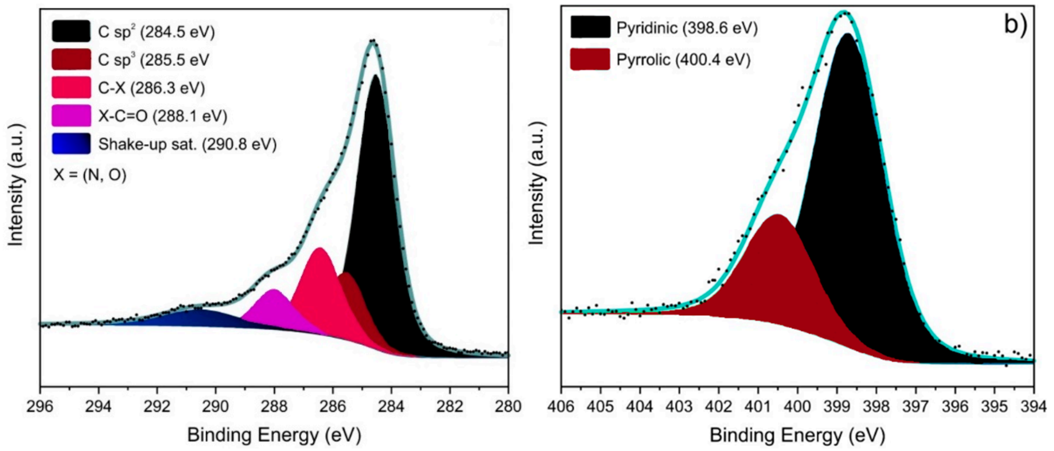

Figure S2: NG not-monochromatic X-ray photoemission spectroscopy (XPS) lines of C1s and N1s, deconvoluted into single chemically shifted components.

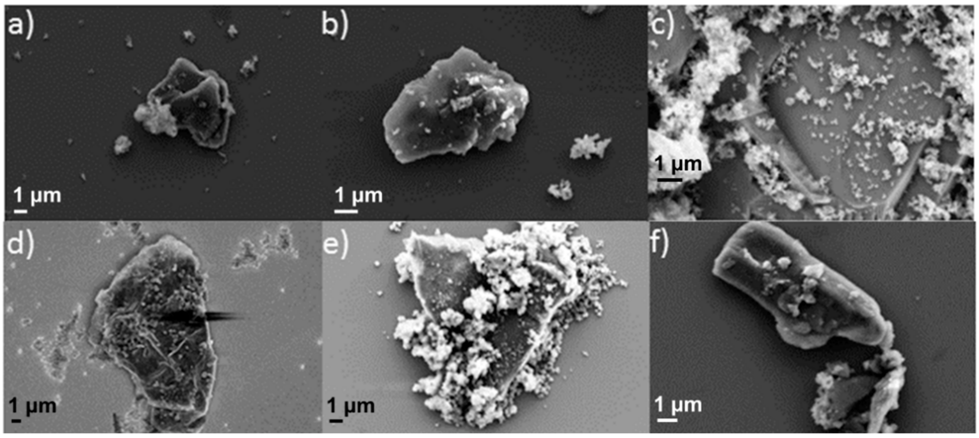

Figure S3. SEM images of as-prepared a) MIL-NG-1, b) MIL-NG-2, c) MIL-NG-3, d) MIL-NG-4, e) MIL-NG-5, f) MIL-NG-6.

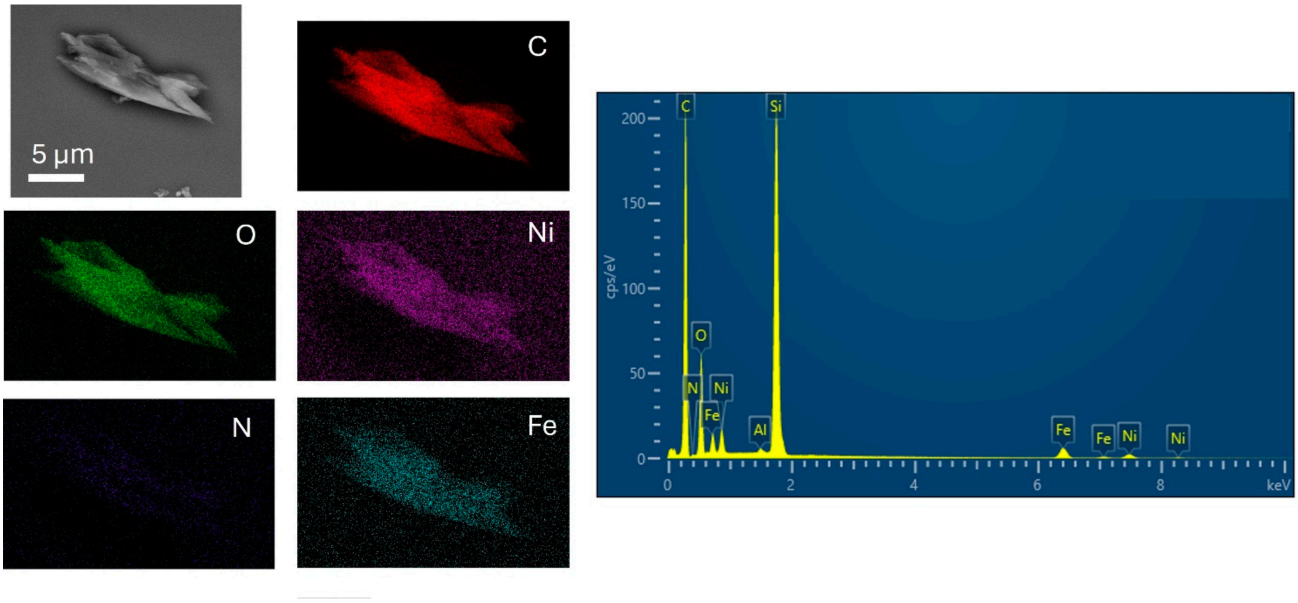

Figure S4. SEM EDX mapping of MIL-NG-3 and EDX spectrum.

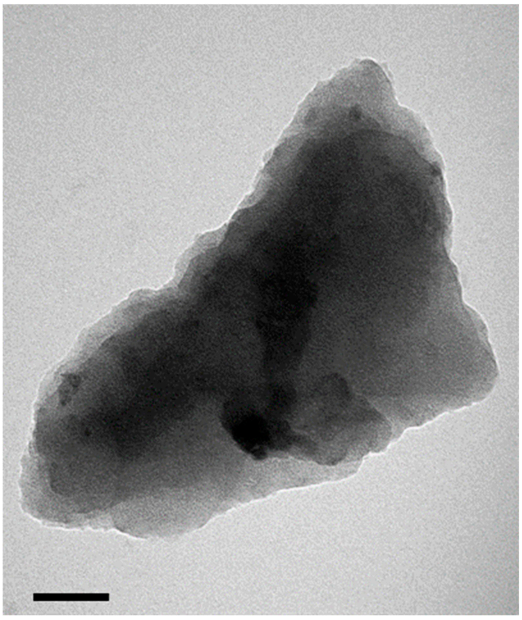

Figure S5: TEM image of NG, scale bar 100 nm.

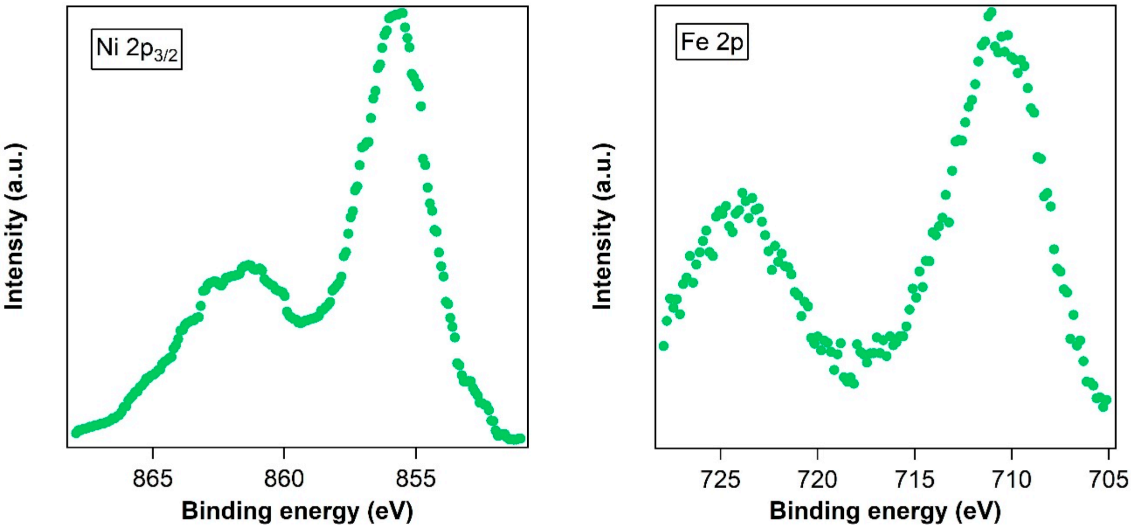

Figure S6: Ni $2p_{3/2}$ and Fe 2p XPS lines of MIL-NG-3 after chronoamperometry in OER indicating the presence of $Ni(OH)_2$/NiOOH and FeOOH.
